# Near Infrared Spectral Linearisation in Quantifying Soluble Solids Content of Intact Carambola

**DOI:** 10.3390/s130404876

**Published:** 2013-04-12

**Authors:** Ahmad Fairuz Omar, Mohd Zubir MatJafri

**Affiliations:** School of Physics, Universiti Sains Malaysia, Penang 11800, Malaysia; E-Mail: mjafri@usm.my

**Keywords:** carambola, NIR, soluble solids content, spectral linearisation

## Abstract

This study presents a novel application of near infrared (NIR) spectral linearisation for measuring the soluble solids content (SSC) of carambola fruits. NIR spectra were measured using reflectance and interactance methods. In this study, only the interactance measurement technique successfully generated a reliable measurement result with a coefficient of determination of (*R^2^*) = 0.724 and a root mean square error of prediction for (RMSEP) = 0.461° Brix. The results from this technique produced a highly accurate and stable prediction model compared with multiple linear regression techniques.

## 1. Introduction

Various statistical analyses have been applied to the measured spectra for the intrinsic quality assessment of fruits to produce higher efficiency prediction algorithms. The current study presents a novel application of near infrared (NIR) spectral linearisation in quantifying soluble solids content (SSC) of carambola (starfruit, *Averrhoa carambola*) fruits. The spectral linearisation technique was originally applied by Omar and MatJafri [1] in monitoring apple and pear decay, as well as by Omar *et al.* [2] in quantifying glucose and fructose aqueous solutions. The results from this study are compared with that obtained from multiple linear regression (MLR) to prove the efficiency of the new technique. MLR is one of the established spectral evaluation techniques, and has been widely applied in spectroscopic analysis for fruit quality measurement. For example, Temma *et al.* [3] and Ventura *et al.* [4] applied MLR for SSC measurement in apples, Abebe [5] for SSC measurement in watermelon, Jaiswal *et al.* [6] for SSC and pH measurement in mangoes, and Jha *et al.* [7] for SSC and pH measurement in banana. The spectral linearisation technique compares the spectral linearity at a predefined range with the actual SSC values measured using a refractometer, unlike MLR, which uses selected wavelengths for prediction algorithm development. In the current study, the NIR wavelength range used for spectral linearisation analysis was between 940 nm and 1,025 nm. This entire selected wavelength range is within the strong water absorbance curve. Water spectrum, with peak absorption at 970 nm, was due to the second O–H stretching band overtone [8,9]. According to Tsenkova [10] and Buning-Pfaue [11], water is the main problem in spectroscopy analysis of other molecules in biological systems. However, new studies on the behaviour of water in biological and aqueous systems have been conducted [12]. Aquaphotimics is a new research field that describes the dynamic spectroscopy of biological and aqueous systems based on water behaviour [10]. The current study implements this concept by interpreting SSC value through the changes in water absorbance patterns. Meanwhile, wavelengths between 900 nm and 930 nm are important for SSC measurement [13], where the wavelength at 910 nm is related to the third C-H stretch overtone [14]. The application of spectral linearisation in quantifying the SSC of carambola has produced the highest accuracy for a prediction model compared with the results analysed using the MLR technique.

## 2. Materials and Methods

**Figure 1.**
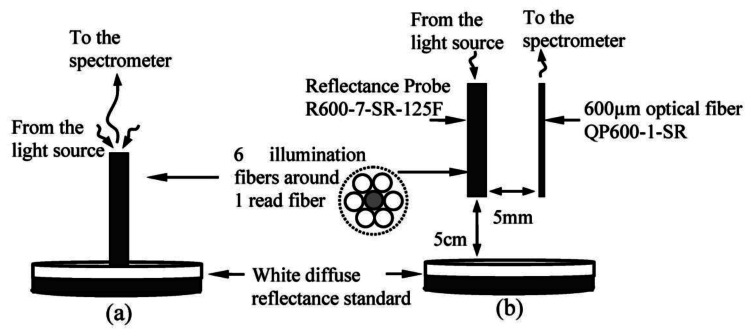
Probe configuration for (**a**) Reflectance calibration setup (**b**) interactance calibration setup.

NIR spectra used in this study were measured using a Jaz Spectrometer (Ocean Optics Inc., Dunedin, FL, USA), with effective wavelengths between 700 and 1,100 nm and optical resolution of ∼0.3 to 10.0 nm (FWHM). A tungsten halogen lamp with spectral emissions between 360 nm to 2,000 nm was used as light source. Two measurement techniques were used in to compare and define the measurement technique that can generate the most reliable prediction model. The first technique is reflectance measurement using a standard reflectance probe with six illumination fibers around one read fiber. Each fiber has a core diameter of 600 μm. The second technique is interactance mode, where the light source and detector are positioned next to each other so that the light due to specular reflection cannot directly enter the detector. By definition, in the reflectance measurement, the field of view of the light detector includes parts of the fruit surface directly illuminated by the source while in the interactance measurement; the field of view of the detector is separated from the illuminated surface by a light seal in contact with the fruit surface [15]. The fiber configurations for reflectance and interactance calibration are shown in Figure 1. The emitting fiber bundle from the reflectance probe was used in the interactance configuration, whereas the retrieving fiber was left unused. The resultant light signal retrieved from the fruit was collected using a single-core fiber with a core diameter of 600 μm, which was placed parallel to the reflectance probe. During the calibration measurement, the interactance probe was located about 5 cm perpendicular to the top surface of the white diffuse reflectance standard, as shown in Figure 1(b). The probe was located directly on top of the fruit sample during the fruit sample measurement for both measuring techniques.

Two data sets were retrieved from different sides of each carambola sample. One set was used for calibration algorithm development and the other was used as prediction sample set. The SSC of carambola juice was measured using the PAL-3 refractometer (Atago, Co., Tokyo, Japan), with a measurement range of 0° Brix to 93° Brix, a resolution of 0.1° Brix, and an accuracy of ±0.2° Brix. Table 1 list the characteristics of the carambola samples. The entire experiment was conducted in a constant laboratory temperature of 23° Celsius.

## 3. Results and Discussion

**Figure 2.**
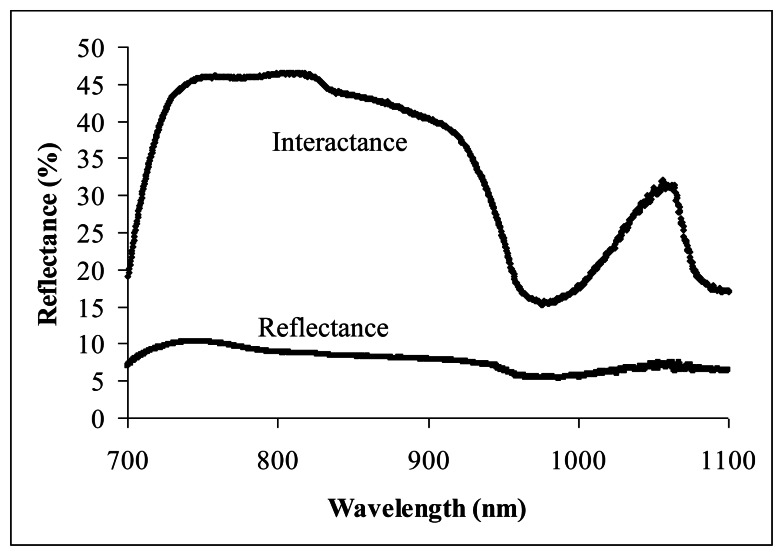
NIR reflectance and interactance spectra of an intact carambola.

Figure 2 shows the NIR spectra obtained through the reflectance and interactance measurement techniques from an intact carambola sample with 7.2 °Brix of SSC. The wavelength of 920 nm was the starting point, where the water absorbance increased rapidly until reaching the peak (bottom reflectance) at about 975 nm. A wavelength of 1,020 nm lies halfway before the NIR moved out from the water absorbance curve at a longer wavelength. Interactance technique has clearly shown the water absorbance curve on the NIR spectrum compared to reflectance technique. This is the main reason interactance technique has produced a much higher correlation in predicting carambola SSC.

**Figure 3.**
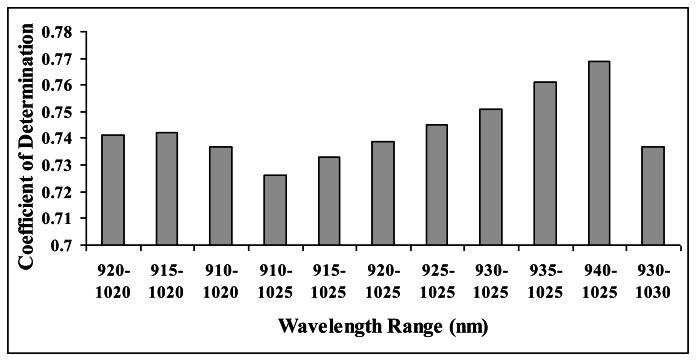
Calibration accuracies from different range of wavelength conducted on interactance spectra.

In finding the best range of wavelength to perform spectral linearisation, a brief statistical approach has been conducted on interactance spectra. Figure 3 show the coefficient of determination obtained when spectral linearisation was performed using different range of wavelength in calibrating carambola SSC. From the wavelength ranges selected for the analysis, wavelengths between 940 nm and 1,025 nm produced the best calibration accuracy (R^2^= 0.769). Hence, detailed study on the development of algorithm in predicting carambola SSC has been conducted by using this wavelength range.

**Figure 4.**
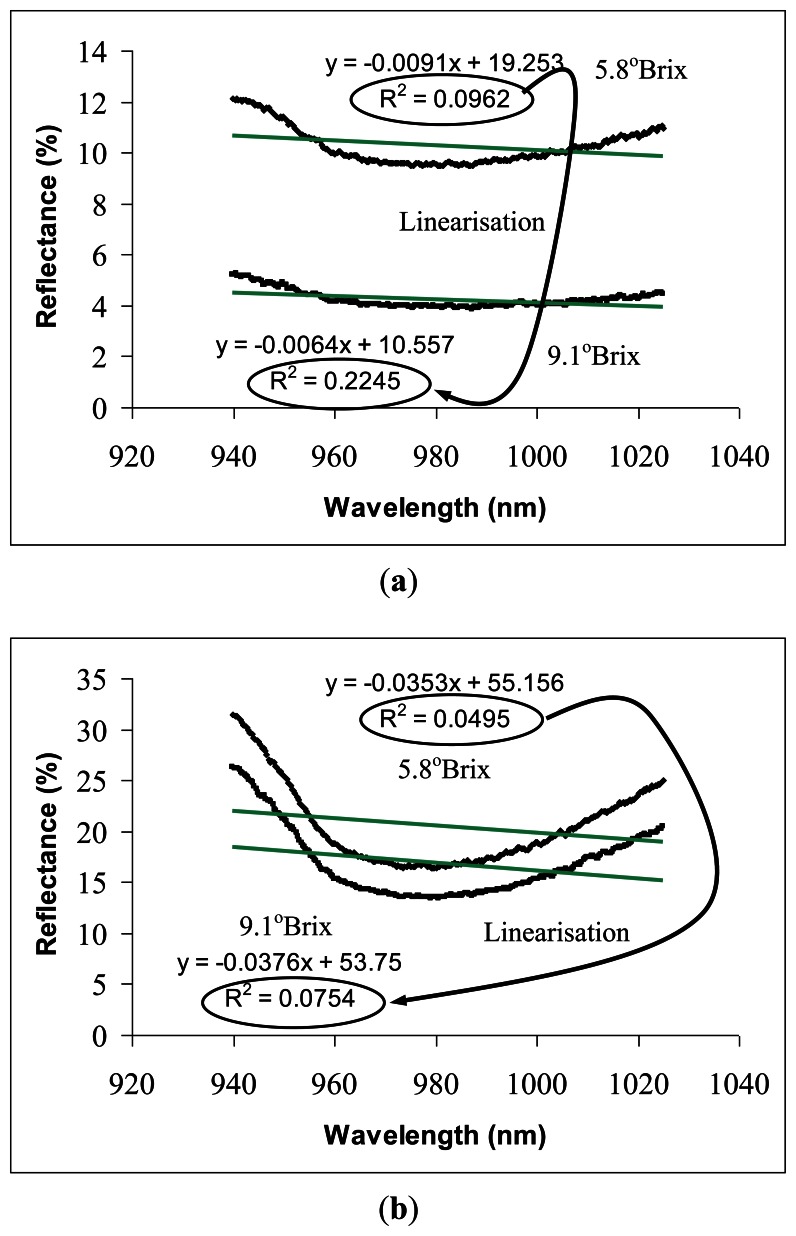
Spectra for two different levels of carambola SSC measured through (**a**) Reflectance (**b**) Interactance.

Figures 4 illustrate the technical method of spectral linearisation on reflectance and interactance spectra. Both figures show that the spectra from the low SSC sample (unripe fruit) was located completely above the reflectance from the sample with high SSC (overripe fruit). The spectra pattern indicates that this scenario results from the combination of specular and diffuse reflectance from the samples with different surface firmness levels. Fully ripened fruits with softer surfaces allow more light penetration through the sample, hence, reducing the reflectance simultaneously at all wavelengths.

Carambola with lower SSC value exhibited a steeper spectral absorbance curve compared with those with higher SSC (Figure 4). SSC (in °Brix) is the percentage of sugars and other soluble solids in water, therefore, a steeper absorbance curve refers to higher water content *per* aqueous fruit sample volume (juice). Spectral steepness is directly associated with the absorbance curve linearity; thus, allowing SSC to be evaluated quantitatively through a technique called spectral linearisation. Spectral linearisation is defined by the value of the linear coefficient of determination, *R^2^* generated from each spectrum. For instance, the linearity of reflectance spectrum increased from 0.0962 to 0.2245 with increased SSC from 5.8° Brix to 9.1° Brix.

Equations (1) and (2) explain the relationship between spectral linearity obtained from the calibration data set and carambola SSC through reflectance and interactance spectra, respectively. This step evaluates the ability of the developed algorithms in producing consistent measurement accuracy levels. The interactance technique produces significantly higher linear coefficient of determination (*R^2^* = 0.769) and lower root mean square of error (RMSEC = 0.422° Brix) compared with the reflectance technique (*R^2^* = 0.614; RMSEC = 0.545° Brix):

(1) SSC(B°rix)=5.05+17.2(R2940−1025)

(2) SSC(B°rix)=2.66+80.9(R2940−1025)

**Figure 5.**
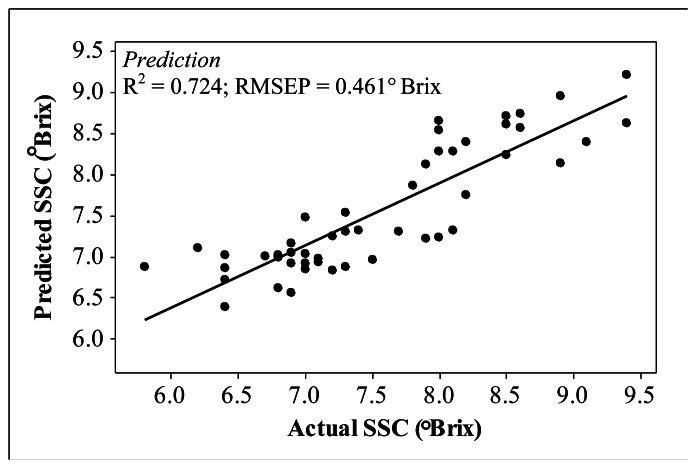
Prediction of carambola SSC through interactance spectral linearisation.

The relationship between the predicted and actual carambola SSC via the interactance technique is illustrated in Figure 5. The interactance measurement technique sustains high accuracy levels in predicting carambola SSC with *R^2^*= 0.724 and root mean square error of prediction (RMSEP) = 0.461° Brix, whereas the reflectance technique produces poor prediction accuracy with R2 = 0.459; RMSEP = 0.645° Brix.

In the application of interactance spectral linearisation for carambola SSC measurement, the technique significantly improved the NIR ability to quantifycarambola SSC. The improvement was from the developed high accuracy prediction model compared with those conducted through MLR, an established statistical method for spectroscopy analysis. Table 2 shows the two other sets of results which were obtained using the MLR technique and also MLR technique with first derivative and Savitzky-Golay smoothing technique on visible and NIR spectroscopy data. Data are usually derivatized to remove background noise from spectra, for example specular light reflection [16]. Besides, Savitzky-Golay smoothing is also one of the methods often used to eliminate noises from spectra [17]. Visible wavelengths were included in the comparative analysis because NIR data alone does not produce any significant results (*R^2^*< 0.4). The failure analysis of NIR interpretation using conventional MLR is expected due to high specular reflectance from the glossy fruit surface. The calibration results using MLR showed even better linearity compared with the spectral linearisation technique, having prediction results with much lower accuracy.

## 4. Conclusions

This study successfully highlighted the importance of spectral linearisation as an alternative technique in quantifying SSC of carambola fruits. Only the interactance measurement technique managed to generate a reliable prediction algorithm with *R^2^*= 0.724 and RMSEP = 0.461° Brix; producing higher prediction accuracy compared with MLR. The application of MLR in this study requires the addition of visible wavelength ranges to generate a useful prediction model. Future research using the spectral linearisation technique should focus on the development of a mathematical representation of the technique and the application of this technique on different fruit types with larger sample size; particularly those with broader SSC ranges.

## Figures and Tables

**Table 1.** Carambola samples used in the experiment.

**Sample**	**Range**		

	**n**	**Weight (g)**	**SSC (° Brix)**
Carambola (B10)	50	95.45–206.19	5.8–9.4

**Table 2.** Results obtained through MLR on visible and NIR spectroscopy data.

**Wavelength (nm)**	**Spectral Treatment**	**Methods**	**Calibration**		**Prediction**	
	
			**R^2^**	**RMSEC**	**R^2^**	**RMSEP**
940–1,025	Spectral Linearisation	Reflectance Interactance	0.614 0.769	0.545 0.422	0.459 0.724	0.645 0.461

450, 470, 580, 615, 650, 670, 730, 740, 911, 950, 970	Multiple Linear Regression	Reflectance Interactance	0.860 0.782	0.369 0.460	0.656 0.702	0.515 0.479

493, 548, 562, 702, 953	First Derivative + Savitzky-Golay, Multiple Linear Regression	Reflectance Interactance	0.562 0.739	0.606 0.468	0.346 0.659	0.709 0.513

## References

[1] Omar A.F., MatJafri M.Z. (2008). Analysis of NIR spectral reflectance linearization and gradient shift in monitoring apple and pear decay. AJOFAI.

[2] Omar A.F., Atan H., MatJafri M.Z. (2011). Application of NIR spectral absorbance linearization and gradient shift in quantifying aqueous glucose and fructose solutions. Asian J. Chem..

[3] Temma T., Hanamatsu K., Shinoki F. (2002). Measuring the sugar content of apples and apple juice by near infrared spectroscopy. Opt. Rev..

[4] Ventura M., de Jager A., de Putter H., Roelofs F.P.M.M. (1998). Non-destructive determination of soluble solids in apple fruit by near infrared spectroscopy (NIRS). Postharvest Biol. Technol..

[5] Abebe A.T. (2006). Total sugar and maturity evaluation of intact watermelon using near infrared spectroscopy. J. Near Infrared Spectrosc..

[6] Jaiswal P., Jha S.N., Bharadwaj R. (2012). Non-destructive prediction of quality of intact banana using spectroscopy. Sci. Hortic..

[7] Jha S.N., Jaiswal P., Narsaiah K., Gupta M., Bhardwaj R., Kumar S.A. (2012). Non-destructive prediction of sweetness of intact mango using near infrared spectroscopy. Sci. Hortic..

[8] Luck W.A.P., Luck W.A.P. (1974). Infrared Overtone Region. Structure of Water and Aqueous Solutions.

[9] Nicolai B.M., Beullens K., Bobelyn E., Peirs A., Saeys W., Theron K.I., Lammertyn J. (2007). Nondestructive measurement of fruit and vegetable quality by means of NIR spectroscopy: A review. Postharvest Biol. Technol..

[10] Tsenkova R. (2010). Aquaphotomics: Water in the biological and aqueous world scrutinised with invisible light. Spectrosc. Eur..

[11] Buning-Pfaue H. (2003). Analysis of water in food by near infrared spectroscopy. Food Chem.

[12] Jinendra B., Tamaki K., Kuroki S., Vassileva M., Yoshida S., Tsenkova R. (2010). Near infrared spectroscopy and aquaphotomics: Novel approach for rapid *in vivo*diagnosis of virus infected soybean. Biochem. Biophys. Res. Commun..

[13] Martinsen P., Schaare P. (1998). Measuring soluble solids distribution in kiwifruit using near-infrared imaging spectroscopy. Postharvest Biol. Technol..

[14] Subedi P.P., Walsh K.B., Owens G. (2007). Prediction of mango eating quality at harvest using short-wave near infrared spectrometry. Postharvest Biol. Technol..

[15] Schaare P.N., Fraser D.G. (2000). Comparison of reflectance, interactance and transmission modes of visible-near infrared spectroscopy for measuring internal properties of kiwifruit (*Actinidia. chinensis*). Postharvest Biol. Technol..

[16] Cen H., He Y. (2007). Theory and application of near infrared reflectance spectroscopy in determination of food quality. Trends Food Sci. Technol..

[17] Herrera J., Guesalaga A., Agosin E. (2003). Shortwav-near infrared spectroscopy for non-destructive determination of maturity of wine grapes. Meas. Sci. Technol..

